# Everybody's Page

**Published:** 1888-02-11

**Authors:** 


### EVERYBODY'S PAGE.

A MAN might as well sleep in his boots as seek repose in a night-cap. Except in very rare cases this article of dress is entirely superfluous, even when not injurious.

THE *Provincial Medical Journal* suggests the application of a solution of sulphide of calcium to mature the eruption in cases of small-pox and to give ease to the patient, and quotes several cases—one of them a very severe one of confluent small-pox—where it has been used with great benefit.

THE first Turkish bath naturalised amongst us was built at the famous town of Blarney, in Ireland, by Mr. Urquhart, in 1856, and this was followed in England by Mr. Crawshay's bath in 1857. Who could count the number that have been built during the thirty years that have elapsed since then!

WHY have the natives of the warmer temperate and torrid zones dark-coloured eyes? "Because they have," would be the reply of Wendell Holmes's "That Boy;" and perhaps of some other people. But science is not content with such dogmatic explanations, and oculists have a theory of their own. It is that the large amount of pigment in dark eyes absorbs the strong light of the sun, and thus acts as a provision of Nature against inflammation of the retina.

THE science of adulteration is evidently getting on well. A machine for mixing oleo-margarine oil with milk, for the making of enriched skim-milk cheese, was exhibited at the recent Dairy Show. It has been used for that purpose extensively at Glasgow, and it will improbably be necessary before long to extend the Margarine Act to cheese. A more creditable use of the machine is that of mixing fat or oil with skim-milk for calves.

A CURIOUS custom of Chinese pisiculturists is mentioned by P. Gresier. They collect the eggs of fishes as they are found floating on the surface of rivers. These are placed in an empty egg-shell, and this is closed and put under a sitting hen with other eggs. When the little chickens are hatched, the egg-shells containing the fish-spawn are emptied into the rivers when the water is heated by the sun, and the young fish soon thrive.

HAIR, as an article of commerce, varies greatly in price. Grey, golden, and auburn are the most valuable colours. The value of hair increases very rapidly in proportion to its length. Thus, while eight-inch hair sells at about a shilling an ounce, hair thirty-six inches long realises as much as thirty shillings an ounce, and all lengths beyond this command fancy prices. The light colours are furnished chiefly by Germany and Austria, and the south of France supplies the darker shades in greatest abundance.

WE are often told that money is the root of all evil—a misquotation, by-the-bye; it is the love of money that St. Paul condemns—but most people will risk a good many moral dangers for the sake of possessing it. When the danger is physical they might be more careful, if they guessed the mischief that may lurk about the coins they handle. Money may easily become the means of spreading disease, though few people think of this as a possible source of infection. Dr. S. Miller Reid declares that the very smell of it has enabled mail-robbers to discover the presence of money in letters. We have heard before now of millionaires who "smelt of gold," but we have always taken the expression as figurative. What if it should be something more? Scarlatina and small-pox may be contracted through touching infected coin, and there is on record the case of a bank official who lost his life through a disease contracted by moistening his fingers at his mouth while counting bank-notes.

IT is said the colour-sense improves by practice, and it is possible that some day practice, aided by heredity, will enable mankind to perceive even the ultra-violet rays our present sight can take no cognisance of. It is certain that women suffer less from colour-blindness than men—presumably from the greater attention they pay to their dress, and to decoration generally.

THE science of electricity has already been pressed into the service of fashion. When at a dance or dinner party a glittering dewdrop seems suddenly to sparkle among the flowers on a lady's shoulder, or a diamond-like flare draws attention to her pretty coiffure, the admiring observer may take it for granted that the possessor of the dewdrops and the starry light in her hair is secretly pressing a tiny battery, ingeniously concealed about her person, by means of which the electric spark flies up, to the danger perhaps of the beholder, if not of the possessor.

QUILLAI BARK is superior to soap for cleansing the hair. This bark contains a neutral vegetable principle, scientifically known as saporine, which has most of the properties of soap. It is found in the common soap wort, in Senega root, and in several lichens, but most abundantly in the inner bark of the *Quilleya saponæia*, or quillai, a small evergreen shrub which is found in Chili. The Chilian women use it for washing the hair, which it is said to improve greatly. A small piece of the bark stirred in cold or hot water will be found to yield a copious lather.

THE sabot of provincial France, harsh and noisy as it is, is less unhealthy than the boot with an absolutely rigid sole which is mostly worn by the English bucolic, whose ungraceful gait is largely due to his being shod in a fashion that prevents the natural play of foot and toes. The sabot has a curve to its sole that allows of a rolling movement of the foot not unlike its natural bending. Moreover, it is generally loose enough to allow of considerable play of the foot and ankle-joint in the act of walking. It is also lighter than the boot of the so-called clodhopper; so for a solid and substantial foot-covering it combines many advantages.

THE popular regard for woollen underclothing may go too far when it leads ordinary healthy people to prefer wool to cotton or linen for night attire. Either of the latter is better than wool, inasmuch as it gives rest to the skin, which may have been unduly stimulated by woollen under-garments during the day. The night-dress is worn under conditions that favour inactivity of the functions of the skin, and is therefore little concerned in the process of evaporation. Moreover, it is not worn for warmth, which is supplied by the bed-clothes; and, except for those who are rheumatic, and for the old and the very young, where the animal heat is but feebly maintained, it is better to employ a material which will give the skin needed rest.

WITH a large fire in a sick-room, there is no need to be afraid of supplying air too freely. Three thousand cubic feet per hour has been fixed as the amount required for a healthy adult; it is obvious that a sick adult should have more than this. Four thousand five hundred cubic feet should be allowed in an ordinary or surgical hospital when there are many bad cases. For a patient suffering from an infectious disease it is desirable to try to obtain six thousand cubic feet per hour. To get this amount without draught, when no mechanical contrivance is available, the capacity per head of the sick-room should be about two thousand cubic feet—the air space now officially required per head in infectious wards.

